# Oregon State Dental Society

**Published:** 1874-06

**Authors:** G. W. Gray


### Proceedings of Societies.

### OREGON STATE DENTAL SOCIETY.

The Oregon State Dental Society met at Odd Fellows Temple in the city of Portland March 25th, 1874, at 10 o'clock A. M. The President, Dr. G. N. Hatch, in the chair.

### AFTERNOON SESSION.

The Society met at 2 P. M., and was called to order by the President.

The minutes of the previous meeting were read and approved.

The reading of theses being in order, Dr. Wm. F. Thompson read a paper before the society, on improvements in operative Dentistry which was well received.

Dr. J. R. Cardwell being called, read a paper on "Alveolar Abscess."

The subject was taken up for general discussion, in which Drs. Gray, Thompson, Skiff, E. O. and H. Smith, Glenn and others participated at considerable length and to the interest of all parties present.

The Society then adjourned until 7 o'clock P. M.

### EVENING SESSION.

The Society met at 7 P. M., and was called to order by the President.

Dr. Gray, being called upon, read a thesis on "Pathology and Therapeutics," in which he referred to an anomalous and interesting case, and requested discussion and the opinion of the Society as to the most effective mode of treatment.

The subject was accordingly taken up, and an interesting discussion followed, in which numerous members participated.

Dr. Wm. H. Koehler then read a paper, subject, "Why do teeth decay?" it was thoroughly discussed, and various opinions entertained as to the probable cause.

On motion, the Society adjourned until 10 o'clock A. M., the following day.

### 2D DAY, MORNING SESSION.

The Society met at 10 o'clock A. M., pursuant to adjournment.

The annual election of officers being the next order of business, the vote was taken, and the following officers were elected to serve during the ensuing year: President, Dr. William F. Thompson; Vice President, Dr. J. R. Cardwell, Recording Secretary, Dr. G. W. Gray; Corresponding Secretary, Dr. Thomas L. Nicklin; Treasurer, Dr. John Welch.

A resolution introduced by Dr. Koehler, thanking the retiring officers for the fair and impartial manner in which they had discharged their various duties, was adopted by a unanimous vote.

Dr. Thompson, on being conducted to the chair, made a neat little speech, thanking the members for the honor bestowed upon him in electing him to so responsible a position, and pledging himself to discharge the duties thereof to the best of his ability.

The reading of theses being again in order, Dr. Thos. L. Nicklin was called on, and read a paper on "Neuralgia: Its causes and treatment." The subject was afterwards generally discussed by the Society. Dr. E. Biddle, of Corvallis, then read a treatise on "Deciduous teeth and their treatment."

Dr. J. H. Hatch, of this city, being called upon, read a paper on "Sensitive Dentine."

The society then adjourned to meet at Dr. Hatch's office at 2 P. M., when Dr. Hatch demonstrated his method of operating by filling a difficult cavity in buccal surface of the second left inferior molar.

The Society then took a recess until 8 o'clock P. M.

### EVENING SESSION.

The Society was called to order at 8 o'clock P. M., by the President, Dr. William F. Thompson.

Dr. E. O. Smith presented an appliance for taking an articulation, which was referred to the Committee on Mechanical Dentistry, who reported favorably thereon.

Dr. H. Smith presented an improved dental plate, which was also submitted to the same committee, who were favorably impressed, but desired further time before making a full report.

The remainder of the evening was devoted to the discussion of various subjects of interest to the profession.

On motion of Dr. Hatch, a vote of thanks tendered to the Trustees of the Odd Fellows' Temple for the use of the same during the meeting was unanimously adopted.

On motion of Dr. Gray, the Society then adjourned to meet at Albany the first Wednesday in June, 1875.

G. W. GRAY, Secretary.

